# Cutaneous vascular calcifications in patients with chronic kidney disease and calcific uremic arteriolopathy: a cross-sectional study

**DOI:** 10.1007/s40620-023-01707-8

**Published:** 2023-07-19

**Authors:** Anne Kristine Røndbjerg, Mette Gyldenløve, Dorrit Krustrup, Marianne Rix, Ilse Vejborg, Lars Lonn, Niklas Rye Jørgensen, Andreas Pasch, Lone Skov, Ditte Hansen

**Affiliations:** 1 Department of Internal Medicine, University Hospital Zealand, Roskilde, Denmark; 2 Department of Nephrology, Copenhagen University Hospital, Rigshospitalet, Denmark; 3 Department of Nephrology, Copenhagen University Hospital, Herlev, Denmark; 4 Department of Dermatology and Allergy, Copenhagen University Hospital, Gentofte, Denmark; 5 Department of Pathology, Copenhagen University Hospital, Herlev, Denmark; 6 Department of Breast Examinations, Copenhagen University Hospital, Gentofte, Denmark; 7 Department of Radiology, Copenhagen University Hospital, Rigshospitalet, Denmark; 8 Department of Clinical Biochemistry, Copenhagen University Hospital, Rigshospitalet, Denmark; 9 Calciscon, Biel, Switzerland; 10 Department of Physiology and Pathophysiology, Johannes Kepler University Linz, Linz, Austria; 11 Department of Clinical Medicine, University of Copenhagen, Herlev, Denmark; 12 Department of Nephrology, Herlev and Gentofte Hospital, University of Copenhagen, Borgmester Ib Juuls Vej 1, 2730 Herlev, Denmark

**Keywords:** Calcific uremic arteriolopathy, Chronic kidney disease, Calcifications, Calcification propensity

## Abstract

**Introduction:**

Calcific uremic arteriolopathy is a life-threatening cutaneous condition in patients with chronic kidney disease. Often, clinical diagnosis is accompanied by histopathologic evaluations demonstrating vascular calcium deposits. We aimed to investigate the presence of cutaneous calcifications in non-lesional tissue in patients with chronic kidney disease, and the relation to systemic vascular calcification.

**Methods:**

We investigated the presence of cutaneous vascular calcifications in non-lesional skin biopsies from patients with current or previous calcific uremic arteriolopathy and patients with different stages of chronic kidney disease without calcific uremic arteriolopathy, and explored their association with vascular calcification in other vascular beds. Systemic vascular calcification was examined by mammography and lumbar X-ray.

**Results:**

Thirty-nine adults were enrolled (current or previous calcific uremic arteriolopathy, *n* = 9; end-stage chronic kidney disease, *n* = 12; chronic kidney disease stage 3b-4, *n* = 12; healthy controls, *n* = 6). All calcific uremic arteriolopathy patients had end-stage kidney disease. Cutaneous vascular calcifications were not present in any of the non-lesional skin punch biopsies. Breast arterial calcification was demonstrated in patients with calcific uremic arteriolopathy (75%) and chronic kidney disease (end-stage 67% and stage 3b-4 25%, respectively), but in none of the controls. All chronic kidney disease patients had systemic calcification on lumbar X-ray (median score 21, 22, and 15 in patients with calcific uremic arteriolopathy, end-stage kidney disease and chronic kidney disease stage 3b-4). The serum calcification propensity was significantly different between groups.

**Discussion:**

Despite a high burden of systemic vascular calcification, cutaneous calcium deposits in non-lesional tissue could not be demonstrated histopathologically in patients with chronic kidney disease (with or without current or previous calcific uremic arteriolopathy). Further studies to determine whether these findings are representative or attributed to other factors are warranted.

**Graphical abstract:**

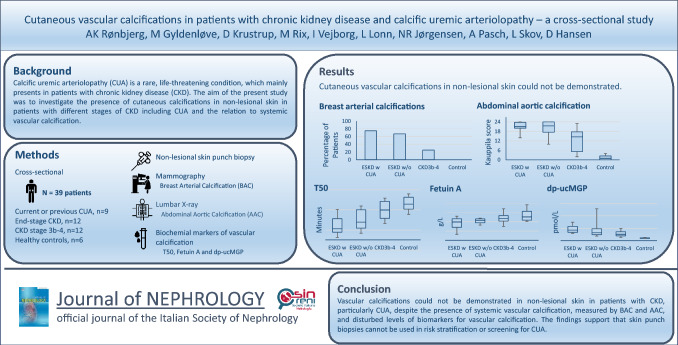

## Introduction

Calcific uremic arteriolopathy (CUA), also known as calciphylaxis, is a rare, life-threatening condition, which mainly occurs in patients with chronic kidney disease (CKD). Initially, patients typically present with rather unspecific skin manifestations, which rapidly progress into multiple, intensely painful, ischemic lesions. Sepsis is the most common cause of death. The pathogenesis of CUA is poorly understood but likely results from an imbalance between calcification promoters and inhibitors [1].

Clinically suspected CUA is frequently supported by histopathologic evaluations. The diagnostic value of biopsies is questioned, as the histopathological changes have also been demonstrated in CKD patients without CUA [2–4]. In addition, skin biopsy is even suggested to propagate or complicate the disease [5].

Outside the setting of CUA, cutaneous calcifications in non-lesional tissue are rarely investigated in patients with CKD. The primary aim of this study was to describe the presence of small-vessel cutaneous calcifications in non-lesional tissue in patients with different stages of CKD, including patients with current or previous CUA. The relation to systemic vascular calcification was examined.

## Methods

### Study protocol and informed consent

Written informed consent was obtained from all participants before screening, and the study was conducted according to the latest revision of the Helsinki Declaration. The protocol was approved by the research ethics committee of the Capital Region of Denmark (H-15003900) and the study was performed in accordance with the ethical standards as laid down in the 1964 Declaration of Helsinki and its later amendments.

### Study subjects

Participants were recruited and sub-grouped based on kidney function: end-stage kidney disease (ESKD) (receiving dialysis) without previous or current CUA (ESKD without CUA), *n* = 12; CKD stage 3b-4 (estimated glomerular filtration rate (eGFR) 15–44 ml/min/1.73 m^2^, not receiving dialysis), *n* = 12, and controls, *n* = 6. In addition, all accessible patients with current or previous CUA were enrolled in the study. All CUA patients had ESKD and received dialysis (ESKD with CUA), *n* = 9.

For patients with CKD, inclusion criteria were age ≥ 18 years and abdominal aortic calcium (AAC) score ≥ 5. In addition, dialysis vintage ≥ 6 months was applied for patients in the ESKD group. For controls, inclusion criteria were age ≥ 18 years, no history of dermatological or kidney disease. For patients with CUA, no other inclusion criteria applied. To examine the presence of cutaneous vascular calcification in lesional biopsies, we obtained and examined skin punch biopsies from 6 patients with clinical CUA (current or previous).

### Skin punch biopsy

A 4 mm non-lesional skin punch biopsy was collected from the lateral thigh. Tissue samples were fixed in neutral buffered formalin and embedded in paraffin wax for histopathology. Skin sections were stained with hematoxylin and eosin (H&E), von Kossa, and Alizarin for detection of calcifications. Histologic examinations were performed by an experienced pathologist (DK) who was blinded to subject characteristics.

### X-ray imaging (mammography and lumbar X-ray)

Unilateral mediolateral oblique mammograms and mammograms including the proximal part of the brachial skin were performed of the craniolateral breast region and corresponding upper arm. Presence of breast arterial calcifications (BACs) were reviewed by an experienced radiologist (IV), who was blinded to subject characteristics.

Lateral lumbar X-ray was performed in the standing position and evaluated by an experienced, blinded radiologist (LL) and a nephrologist (AKR). Abdominal aortic calcification score was calculated according to Kauppila [6] with values ranging from 0 (no abdominal aortic calcification) to 24 (most severe degree).

### Biochemical analyses

Non-fasting blood samples were drawn for routine analyses at the Department of Clinical Biochemistry, Rigshospitalet, University of Copenhagen. In addition, calcification propensity (T50) [7] and Fetuin A [8] were determined by Calciscon, Nidau, Switzerland. In short, using blood samples that had never been thawed before, the calcification propensity, serum T50, was assessed using the method described by Pasch et al. [7]. In order to start the synthesis of primary calcification protein particles, samples were supersaturated by adding calcium (10 mM) and phosphorus (6 mM). Using a nephelostar nephelometer, the time (in minutes) required for spontaneous conversion to secondary calcification protein particles was determined (BMG Labtech, Ortenberg, Germany). The maximum intra-day and inter-day coefficients of variation are 3.3% and 5.4%, respectively. The same specimen was also used to measure fetuin-A. An analysis using a BMI nephelometric test was used. After centrifuging serum samples for 60 min at 15,000×*g*, PBS was added for four-fold dilution (N Diluent, Dade Behring Holdings, Liederbach, Germany). A polyclonal rabbit anti-human fetuin-A antibody was added to serum, as previously reported [9]. Upon agglutination with fetuin-A, scattered light intensity increases. Serum concentrations of fetuin-A are determined by regression analysis of standard curves using a control solution of purified serum fetuin-A powder (Boehringer Mannheim GmbH, Mannheim, Dade Behring, Marburg, Germany). In order to rule out cross-reactivity of the antibodies with other serum proteins and proteolytic fragments of fetuin-A, the assay was compared side by side with immunoblot studies. With fetuin-B, the test does not cross-react. Intra- and inter-assay coefficients of variation are 7.7% and 8.1%, respectively. The assay concentration range is 0.05–3.5 g/L.

Fibroblast growth factor (FGF) 23, calcitriol, bone alkaline phosphatase (BAP), and dephosphorylated, uncarboxylated, fully inactive Matrix Gla protein (dp-ucMGP) (indirect measure of vitamin K status) were analyzed at the Department of Clinical Biochemistry, Rigshospitalet. FGF23 was determined using the Liaison FGF23^®^ assay on the Liaison XL automated analyzer (DiaSorin, Saluggia, IT). Calcitriol, BAP and dp-ucMGP were all analyzed on the IDS-iSYS Multi-Discipline Automated System (Immunodiagnostic systems, plc, Tyne and Wear, UK) using the following assays: Calcitriol was determined using the IDS-iSYS 1,25 VitD^Xp^ assay; BAP was measured using the IDS-iSYS Ostase^®^ BAP assay; dp-ucMGP was determined using the IDS-iSYS Ina*K*tif MGP assay.

All assays were chemiluminescence immunoassays. Assay performance was verified using the manufacturers’ control specimens. The intermediary precisions expressed as coefficients of variation for FGF23 were 5.3% and 4.4% at levels of controls of 117 and 717 ng/L, respectively. For calcitriol the intermediary precisions were 17%, 15%, and 15% at levels of 69, 81, and 179 pmol/L, respectively. For BAP the intermediary precisions were 8.5%, 7.1%, 3.7%, and 6.3% at levels of 4.5, 13.2, 20.1, and 52.1 µg/L, respectively. Finally, for dp-ucMGP the control specimens had average values of 900 pmol/L, 4100 pmol/L, and 7050 pmol/L with intermediary precisions of 3.2%, 2.8%, and 1.6%, respectively.

### Statistics

Continuous data are presented as median and interquartile range. Comparisons between the groups were performed with Kruskal Wallis and Mann–Whitney tests. Categorical data are presented as number and percentage. Chi-square and Fisher’s exact test were used for categorical variables. Due to the small numbers, some of the statistical comparisons were violated and not applicable. *P* values were two-sided, and *P* < 0.05 was considered statistically significant. All analyses were made with SPSS (version 25.0, IBM).

## Results

A total of 39 participants were included (Table 1). Of these, nine with ESKD had CUA (current, *n* = 7; previous, *n* = 2).

**Table 1:** Subject characteristics according to kidney function

	ESKD w CUA^a^ (*n* = 9)	ESKD w/o CUA (*n* = 12)	CKD3b-4 (*n* = 12)	Controls (*n* = 6)	*P* value
Demographics					
Age, years	69 (68–80)	67 (64–74)	73 (62–77)	69 (65–73)	0.706
Female sex	3 (33)	7 (58)	3 (25)	2 (33)	N/A
BMI, kg/m^2^	27 (21–31)	29 (26–31)	29 (28–34)	25 (23–27)	0.084
Hemodialysis	8 (89)	12 (100)	N/A	N/A	
Peritoneal dialysis	1 (11)	0 (0)	N/A	N/A	
Dialysis vintage, months	32 (10–60)	26 (14–46)	N/A	N/A	
Smoking (current or previous)	5 (56)*	12 (100)	9 (75)	4 (67)	N/A
Comorbidity					
Type-2 diabetes	4 (44)	6 (50)	7 (58)	1 (17)	N/A
Hypertension	8 (89)	10 (83)	12 (100)	1 (17)	N/A
Cardiovascular disease^b^	5 (56)	9 (75)	6 (50)	1 (17)	N/A
Current CUA	7 (78)				
Previous CUA	2 (22)				
Current medication					
Vitamin K antagonist	1 (11)	2 (17)	2 (17)	0 (0)	N/A
Calcium-based phosphate binder	2 (22)	7 (58)	1 (8)	0 (0)	N/A
Non-calcium-based phosphate binder	7 (78)	6 (50)	0 (0)	0 (0)	N/A
Cholecalciferol	4 (44)	8 (67)	8 (67)	2 (33)	N/A
Vitamin D analog	7 (78)	9 (75)	3 (25)	0 (0)	N/A
Cholesterol lowering	5 (56)	8 (67)	11 (92)	2 (33)	N/A
Iron supplement	7 (78)	4 (33)	4 (33)	0 (0)	N/A
Punch biopsies					
Calcifications	0 (0)	0 (0)	0 (0)	0 (0)	N/A
X-ray imaging					
AAC score	21 (16–24)	22 (18–24)	15 (5–18)	1(0–3)	0.000
BAC (*n* = 20)	6 (75)^c^	8 (67)	3 (25)	0 (0)	N/A
Biochemical parameters					
ALAT, U/L	15 (11–21)	19 (16–23)	23 (17–29)	26 (22–33)	0.014
Albumin, g/L	30 (27–33)	31 (28–35)	36 (34–40)	39 (37–41)	0.000
Creatinine, µmol/L	740 (482–848)	645 (441–797)	183 (154–231)	80 (71–99)	0.000
eGFR, ml/min1.73m^2^	6 (5–8)	7 (4–8)	28 (23–37)	80 (64–87)	0.000
Ionized calcium, mmol/L	1.23 (0.99–1.24)	1.17 (1.10–1.24)	1.20 (1.16–1.29)	1.22 (1.20–1.23)	0.380
Phosphate, mmol/L	1.81 (1.51–2.19)	1.58 (1.46–1.86)	1.05 (0.81–1.18)	0.91 (0.79–1.00)	0.000
PTH, pmol/L	28 (19–66)	29 (13–43)	11 (8–16)	4 (4–5)	0.001
Mg, mmol/L	0.92 (0.85–0.94)	0.95 (0.89–1.02)	0.82 (0.74–0.89)	0.86 (0.81–0.94)	0.017
BAP, ug/L	34 (17–46)	25 (20–35)	19 (16–21)	19 (16–23)	0.129
FGF23, ng/L	15,420 (5895–65,975)	4750 (2518–12,453)	147 (120–192)	58 (49–74)	0.000
25(OH)D, nmol/L	38 (21–61)*	63 (37–77)	66 (52–95)	95 (75–123)	0.005
Calcitriol, pmol/L	27 (21–34)	29 (26–45)	121 (70–132)	148 (109–221)	0.000
T50, min	110 (76–212)	177 (118–300)	298 (215–384)	357 (307–425)	0.001
Fetuin A, g/L	0.40 (0.28–0.47)	0.43 (0.38–0.46)	0.47 (0.42–0.53)	0.50 (0.44–0.63)	0.051
Dp-ucMGP, pmol/L	1706 (1350–2283)	1280 (976–1929)	1030 (768–1438)	411 (372–449)	0.000

Data are presented as *n* (%) for categorical measures and median (interquartile range) for continuous data

*AAC* abdominal aortic calcification, *ALAT* alanine transaminase, *BAC* breast arterial calcification, *BAP* bone alkaline phosphatase, *BMI* body mass index, *Ca* calcium, *CKD3b-4* chronic kidney disease stage 3b-4 (eGFR 15–44), *CUA* calcific uremic arteriolopathy, *dp-ucMGP* uncarboxylated, dephosphorylated, fully inactive matrix Gla protein, *eGFR* estimated glomerular filtration rate, *ESKD* end-stage kidney disease, *FGF23* fibroblast growth factor 23, *HD* hemodialysis, *Mg* magnesium, *N/A* not applicable, *PTH* parathyroid hormone, *T50* calcification propensity, *w* with, *w/o* without, *25(OH)D* 25-hydroxyvitamin D

^a^Current or previous CUA

^b^Cardiovascular disease = ischemic heart disease, peripheral artery disease, or stroke

^c^Mammography is missing for one patient

*Significantly different (*P* value < 0.05) for ESKD with CUA compared to ESKD without CUA

**Fig. 1:**
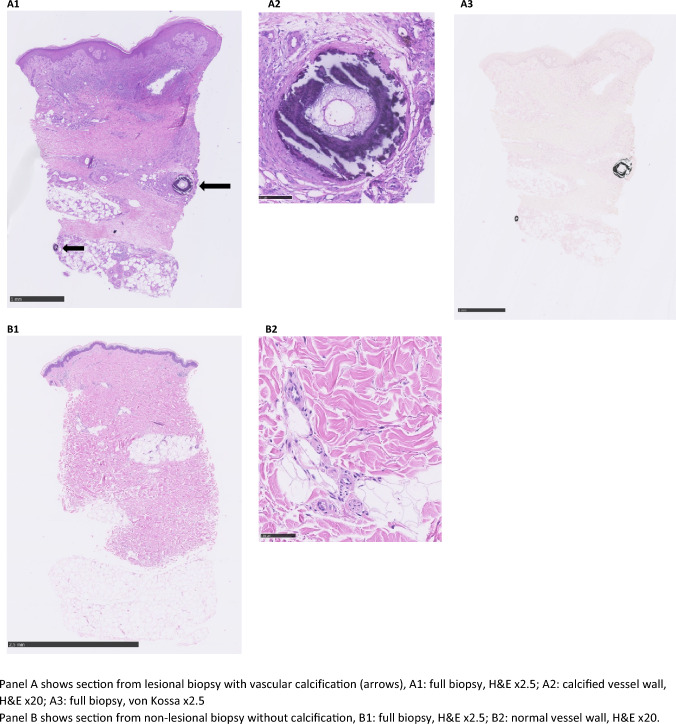
Lesional and non-lesional skin biopsies

Cutaneous vascular calcifications on histopathologic evaluations were not found in any of the non-lesional skin punch biopsies from either CUA patients or the remaining participants with ESKD without CUA, CKD3b-4 or controls (Tables 1 and 2). Cutaneous vascular calcification in a sample of lesional skin punch biopsies from 6 patients with clinical CUA were examined. Vascular calcifications were found in *n* = 3 (Table 2 and Fig. 1).

**Table 2:** Histopathology findings in lesional and non-lesional biopsies

	ESKD w CUA Non-lesional (*n* = 7)^a^	ESKD w CUA Lesional (*n* = 6)^b^	ESKD w/o CUA Non-lesional (*n* = 12)	CKD3b-4 Non-lesional (*n* = 12)	Controls Non-lesional (*n* = 6)
Biopsy material					
Presence of subcutis	6 (87)	6 (100)	10 (83)	11 (92)	5 (83)
Presence of eccrine glands	5 (71)	6 (100)	9 (75)	11 (92)	5 (83)
Histopathology					
Calcification in vessel wall	0 (0)	3 (50)	0 (0)	0 (0)	0 (0)
Calcification in dermis	0 (0)	0 (0)	0 (0)	0 (0)	0 (0)
Perieccrine calcification	0 (0)	0 (0)	0 (0)	0 (0)	0 (0)
Calcification of subcutis	0 (0)	2 (33)	0 (0)	0 (0)	0 (0)
Necrosis	0 (0)	2 (33)	0 (0)	0 (0)	0 (0)
Ulceration	0 (0)	1 (17)	0 (0)	0 (0)	0 (0)
Fat necrosis	0 (0)	2 (33)	0 (0)	0 (0)	0 (0)
Panniculitis	0 (0)	2 (33)	0 (0)	0 (0)	0 (0)

Data are presented as *n* (%)

*CKD3b-4* chronic kidney disease stage 3b-5 (eGFR 15–44), *CUA* calcific uremic arteriolopathy, *ESKD* end-stage kidney disease (receiving dialysis), *w* with, *w/o* without

^a^Non-lesional skin punch biopsies are missing for two ESKD patients with CUA

^b^Lesional skin punch biopsies are missing for three ESKD patients with CUA

**Fig. 2:**
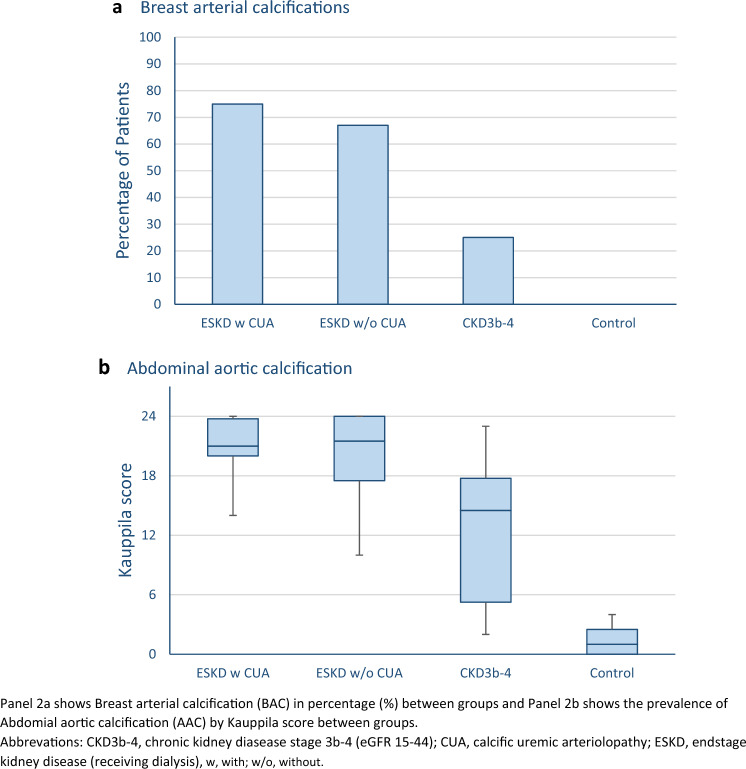
Prevalence of breast arterial calcifications and abdominal aortic calcifications between groups

Small-vessel BACs were present in *n* = 6/8 (75%) in the ESKD with CUA group. In patients with ESKD without CUA and CKD3b-4, corresponding numbers were *n* = 8/12 (67%) and *n* = 3/12 (25%), respectively. None of the control subjects had cutaneous vascular calcifications visible on mammography (Table 1 and Fig. 2a).

All CKD patients had signs of systemic calcification defined by an AAC score ≥ 5. Median AAC score was 21, 22, and 15 in patients with ESKD with CUA, ESKD without CUA, and CKD stage 3b-4, respectively. In control subjects, the median score was significantly lower (AAC = 1). (Table 1 and Fig. 2b).

**Fig. 3:**
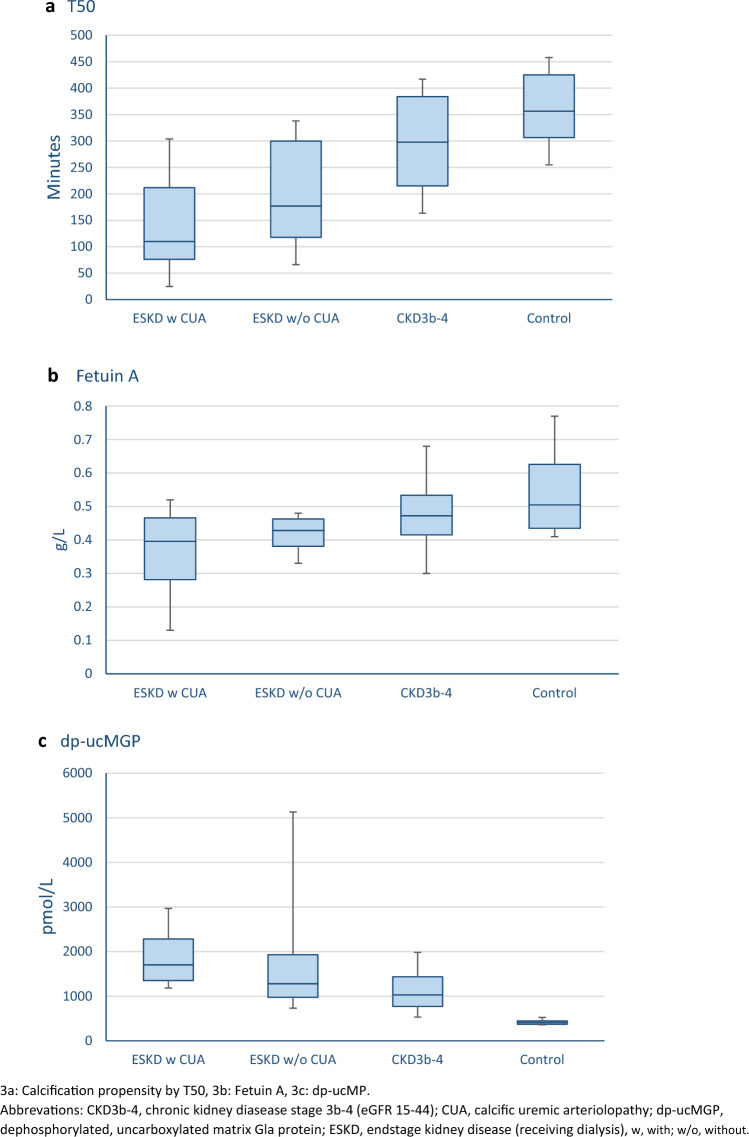
Levels of T50, Fetuin A and dp-ucMGP between groups

Blood levels of parameters related to the mineral metabolism were significantly different between groups (phosphate, PTH, Mg, FGF23, 25(OH)D, calcitriol) but only 25(OH)D showed significant difference when comparing ESKD with and without CUA (Table 1). The calcification propensity score measures time to calcification (T50). T50 appeared shortest and the calcification inhibitor fetuin A appeared lowest in patients with current or previous CUA (Fig. 3a and b) although no statistically significant difference between patients with ESKD with or without CUA were found in this small cohort. The marker of vitamin K deficiency, dp-ucMGP, was also significantly different between groups (Table 1 and Fig. 3c). However, the level of dp-ucMGP did not differ significantly between patients with ESKD with and without CUA.

## Discussion

In the present study, the presence of cutaneous calcifications in non-lesional punch biopsies from patients with ESKD and CKD stage 3b-4 without CUA and ESKD with current or previous CUA were investigated. Despite systemic vascular calcifications, measured by AAC, and higher prevalence of mammographic BACs compared to controls, cutaneous calcium deposits could not be demonstrated histopathologically in non-lesional biopsies from any patients with CKD. Furthermore, no cutaneous calcifications were observed in biopsies from control subjects.

The presence of cutaneous calcifications in patients with CKD and no previous or current CUA has been explored in previous studies. Ellis et al. examined the presence of calcification in marginal tissue from amputation specimens in patients with ESKD and no CUA. Vascular lesions were present in about one third of the specimens [3]. Chaudet et al. also examined tissue from amputation specimens (above-the-knee) and found an overall prevalence of intravascular calcification in 40%, and more prevalent capillary calcifications of 26% for CKD patients (44% ESKD) without CUA (9% for non-CKD patients) [2]. Ruderman et al. sampled full-thickness incisional biopsies from patients with CKD and healthy controls undergoing elective surgical procedures. Vascular calcifications were identified in 38% of the patients with CKD and 18% of the controls [4]. These findings differ greatly from our study with finer biopsies. This might reflect the larger/deeper biopsies available from amputation specimens or incisional biopsies. Although our participants with CKD had systemic vascular calcifications based on AAC and BAC, the vascular calcifications in the setting of an amputation may indeed be more extreme [2, 3].

The presence of cutaneous calcifications in non-lesional biopsies from patients with previous or current CUA has, to our knowledge, not been studied. Although, these patients had the highest prevalence of BACs and shortest T50 and most disturbed levels of the calcification inhibitors fetuin A and vitamin K measured by dp-ucMGP, they did not present with cutaneous calcification in non-lesional biopsies. This may reflect that CUA lesions occur in a regional pattern, and in accordance with the two-step hypothesis, may occur in a sensitized individual in whom they are triggered by challenging factors such as trauma [10]. It may also confirm that cutaneous calcification is not pathognomonic for CUA as addressed in other studies with lesional biopsies from patients with CKD [11].

Vascular calcification occurs at two anatomical sites within the vascular wall, the intima and the media, and is the consequence of two different pathological processes. Medial arterial calcification, first described by Mönckeberg in 1903, is a non-occlusive, circumferential calcification of the media of small-to-medium-sized arteries [12]. Breast arterial calcification is considered a variant of medial vascular calcification [13]. In the present study, BAC was demonstrated in patients with CUA (75%) and CKD (ESKD 67% and stage 3b-4 25%, respectively), and in none of the controls. The prevalence of BAC on mammography varies greatly among published studies, which likely reflects heterogeneity of populations and mammography equipment. BAC is present in up to 70% of patients with CKD and this is in agreement with our results [13]. The present study was not large enough to examine for any significant difference in frequency of BAC between ESKD with and without CUA.

To our knowledge, this study is the first to systematically assess cutaneous calcifications across the spectrum of kidney function in non-lesional biopsies. Strengths are the sampling of biopsies in different CKD stages expected to have a high risk of vascular calcification due to the high AAC and BAC scores. The lateral thigh was chosen for biopsy as CUA typically involves locations with cutaneous and subcutaneous adiposity. Limitations of the study include the cross-sectional design and relatively small sample size. In addition, larger and deeper biopsies, e.g., incisional biopsies, may have allowed for better histopathological evaluations. However, these were avoided to reduce the risk of ulceration especially in patients with current CUA. In planning the study, AAC score ≥ 5 (corresponding to moderate calcification) was chosen as an inclusion criterion for patients with CKD, as these patients were expected to be at high risk of cutaneous vascular calcification. Although all patient groups ended with median AAC scores > 15, the criterion might have biased results, as AAC ≥ 5 was not required in the control group.

Although the role and importance of cutaneous calcifications in CUA is challenged, the present study adds support to the view that patients with CUA have more systemic calcification with increased BAC and lower T50. According to the present findings, however, punch biopsies cannot be used in risk stratification or screening of patients. Further studies are needed to determine whether the current findings are representative or could be attributed to other factors.

## Data Availability

The data may be available on request to the corresponding author.
